# Characterizing mental health related service contacts in children and youth: a linkage study of health survey and administrative data

**DOI:** 10.1186/s13034-022-00483-w

**Published:** 2022-06-21

**Authors:** Jordan Edwards, Li Wang, Laura Duncan, Jinette Comeau, Kelly K. Anderson, Katholiki Georgiades

**Affiliations:** 1 Department of Psychiatry and Behavioural Neurosciences, McMaster University, Hamilton, ON Canada; 2 Offord Centre for Child Studies, McMaster University, Hamilton, ON Canada; 3 Department of Sociology, King’s University College, Western University, London, ON Canada; 4 Department of Epidemiology & Biostatistics, Western University, London, ON Canada; 5 Department of Psychiatry, Schulich School of Medicine, Western University, London, ON Canada; 6 Lawson Health Research Institute, London, ON Canada

**Keywords:** Child and youth mental health, Mental health services, Data linkage

## Abstract

**Background:**

To inform the provision and organization of care, and to improve equitable access to mental health services for children and youth, we must first characterize the children and youth being served, taking into consideration factors related to mental health need. Our objective was to use a population-based survey linked with health administrative data to estimate mental health related contacts and determine socio-demographic correlates, after adjusting for factors related to mental health need.

**Methods:**

Data from the 2014 Ontario Child Health Study (OCHS) were linked at the individual level to health administrative databases from Ontario’s Ministry of Health and Long-Term Care (MOHLTC). Mental health related service contacts were identified in the 6-months prior to the OCHS survey date. Service contacts with physicians were obtained from health administrative data, and non-physician service contacts from survey data (parent-report).

**Results:**

21.7% of Ontarian children (4–11 years) and youth (12–17 years) had at least one mental health related contact in the 6-months prior to their OCHS survey date (18.8% non-physician, 8.0% physician, 5.2% both). Children and youth contacting both physician and non-physician services (ref. contact with physician or non-physician services alone) had higher mean symptom ratings of mental disorders across all classes of disorder. After adjusting for total symptom ratings, children and youth with immigrant parent(s) (ref. non-immigrant) (Prevalence Ratio: 0.65, 95% CI 0.55, 0.75) were less likely to have any mental health related service contact.

**Conclusions:**

Results indicate that children and youth with the highest mental health symptom ratings are more likely to have contact with multiple providers across sectors. As such, the coordination of care across and within sectors are critical components of mental health related services for children and youth. Our results indicate that the greatest disparities in mental health related service contacts may exist for children and youth with immigrant parent(s) and that targeted outreach efforts are required to reduce barriers to care and improve equitable access to mental health related services for children and youth in Ontario.

**Supplementary Information:**

The online version contains supplementary material available at 10.1186/s13034-022-00483-w.

## Introduction

In Canada, mental health services for children and youth is delivered from a wide variety of providers who are funded both publicly and privately [1, 2]. Specifically, all necessary physician visits are covered under public funding, whereas some visits to non-physician providers, such as psychologists in private practice, requires out-of-pocket reimbursement, which may be covered by private health insurance [2]. Children and youth also receive mental health support from school settings, which are universally covered [1, 2]. Perhaps, due in part to the fractured nature of Canada’s mental health care system, Canadian evidence assessing correlates of mental health related service contact among children and youth is fragmented and disparate, arising from varying data sources (survey [1, 3], health administrative data [4, 5]), service sectors (e.g. health, education, social services) in varying geographies (local, regional, provincial). Lack of standardization in measurement and integration of service data from various sectors, including physician and non-physician-based services, has led to methodological challenges and evidence gaps. To inform the provision and organization of mental health services for children and youth, we must first characterize the children and youth being served. Moreover, to increase equitable access to mental health related services, we must identify and characterize disparities in service delivery.

Prior evidence has relied on the use of either health administrative (herein administrative) data or population-level survey data to identify correlates of mental health related service contact [1, 5–7]. Both data sources offer differing strengths and limitations. Administrative data provides broad and continuous coverage of population contacts with physician services, as it was developed to support physician compensation in the Canadian single-payer healthcare system [8, 9]. The major limiting factor of this data source for estimating child and youth mental health related service contacts is the exclusion of non-physician based mental health professionals whose services are not captured [4, 10]. Another important limitation is that, in outpatient settings, clinicians are restricted to one billing code per visit. As such, mental health contacts may be missed when people present with multiple complaints leading to an underestimate of mental health related physician contacts. In contrast, survey data can provide information on contacts with a wider range of mental health professionals and service providers including non-physician services, and more comprehensive data on socio-demographic and clinical factors to support contextualizing the populations being serviced across these various sectors [11]. Furthermore, survey data traditionally offers standardized and culturally sensitive measurement tools [12]. However, survey data relies on self-reported mental health related service contacts, which are subject to recall bias [13–15]. Canada is not alone in its approach to measuring mental health related service contacts among children and youth. Evidence from other nations, including the United States, United Kingdom, and Denmark also relies heavily on estimates derived from either survey or administrative/registry data with varying levels of service coverage and measurement of socio-demographic characteristics and factors related to mental health need [16–21].

One approach to improve current methodology for contextualizing mental health related service contacts among children and youth is to use a population-based survey that has been linked with health administrative data to estimate mental health related service contacts and determine socio-demographic correlates, after adjusting for factors related to mental health need, to identify potential disparities in access to mental health related service sectors. To date, evidence using survey linked data in Canadian settings has focused on adult populations, with a gap existing for children and youth [8]. Our goal was to fill this data gap by leveraging a child and youth focused representative population-level survey and health administrative data linkage [11].

## Methods

### Sample/source of data

#### Survey

The study sample comes from the 2014 Ontario Child Health Study (OCHS), a provincially representative cross-sectional epidemiological survey of children and youth in Ontario, Canada [11]. The sampling frame was the 2014 Canadian Child Tax Benefit file, which covers all children and youth except Indigenous children who live on reserve, homeless youth, and children and youth who are in child welfare and youth justice systems [11]. Evidence suggests this sampling frame is more reliable and efficient than other Census or birth registries [22]. The sampling procedure involved a 3-stage clustered approach with sampling by region and stratification by residency and income [11]. Full details of the study design can be found elsewhere [11].

#### Administrative data

Approximately 89.5% of participants in the OCHS agreed to share and link their individual survey responses with health administrative databases held by the Ontario Ministry of Health and Long-term care (MOHLTC). Linkage was done at the individual-level using deterministic linkage from provincial health card numbers and linked administrative databases contained both retrospective (October 2004, 10-years pre-OCHS) and prospective (September 2018, 3-years post-OCHS) information on all medical services covered under the Ontario Health Insurance Program (OHIP), including inpatient hospitalizations, emergency department visits, and outpatient physician visits, which include visits to family physicians, pediatricians, and psychiatrists. OHIP provides nearly universal coverage of Ontarian residents (> 96%) (See Additional file 1 for description of included databases) [23]. We followed the RECORD guidelines for reporting observational studies using routinely collected data (Additional file 2) [24].

### Mental health related service contacts

#### Non-physician service contacts

Non-physician service contacts were assessed using questions administered to the parent about any contacts in the 6-months prior to the OCHS interview date (included questions in Additional file 3). This includes contacts with individual mental health providers, including psychologists, social workers, other types of counsellors, school guidance counsellors, teachers, or other adults at school and contacts in specialized child and youth mental health settings (CYMHS), focusing on those offering non-physician-based services [25].

#### Physician service contacts

Physician service contacts for a mental health concern in the 6-months prior to the OCHS interview date were assessed using administrative data. This includes contacts with either a family physician, pediatrician, psychiatrist, emergency physician, or other physician specialist. Contacts with a physician can be in either inpatient or outpatient settings. Mental health presentations were identified using diagnostic codes provided by the physician for patient visits (included codes in Additional file 3).

### Contextual factors

#### Factors related to mental health need (mental health symptom ratings)

Mental health symptom ratings were evaluated using the Ontario Child Health Study Emotional Behavioural Scales (OCHS-EBS), which provides dimensional measurement of 7 disorders based on criteria from the Diagnostic and Statistical Manual of Mental Disorders (DSM-5) [26, 27]. We classified mental disorders into three categories; (i) *Internalizing disorders*, which included major depressive disorders (MDD), generalized anxiety disorder (GAD), social anxiety disorder/social phobia (SP), and separation anxiety (SAD); (ii) *Externalizing disorders*, which included oppositional-defiant disorder (ODD), and conduct disorder (CD); and (iii) *ADHD*, which included assessment of attention-deficit hyperactivity disorder (ADHD). The reference period for assessment was the 6-month period prior to OCHS interview date. Items were summed within each category and converted to a standardized score, where 0 represents the mean symptom level in the total sample, and a 1-unit change is indicative of a 1 standard deviation (SD) increase or decrease in symptoms of that class of disorder. The OCHS-EBS has been validated against a structured diagnostic interview, specifically the Mini International Neuropsychiatric Interview for Children and Adolescents (MINI-KID), where it was found to achieve comparable levels of reliability and convergent validity for classifying child mental disorders [27]. The OCHS was administered to both persons most knowledgeable (PMK) about all children (98.6% identified as parents and therefore henceforth are referred to as parent) and youth themselves for ages 12–17. The OCHS-EBS scales can be found online [28].

#### Socio-demographic characteristics

Data on socio-demographic characteristics were obtained from the 2014 OCHS using standardized questions developed by Statistics Canada. These include questions on child age (0 = 4–11; 1 = 12–17), sex (0 = female; 1 = male), number of biological parents in the home (0 = two biological parents; 1 = one or no biological parents), household income below Statistics Canada’s Low-Income Measure (0 = not low-income; 1 = low-income), based on 2013 before tax-cut-offs [29], immigrant parent(s) (0 = both parents born in Canada; 1 = at least one parent born outside Canada), and urbanicity (large centre, small-medium centre, rural area), which was derived from population density and size [30].

### Analysis

#### Sample

**Fig. 1:**
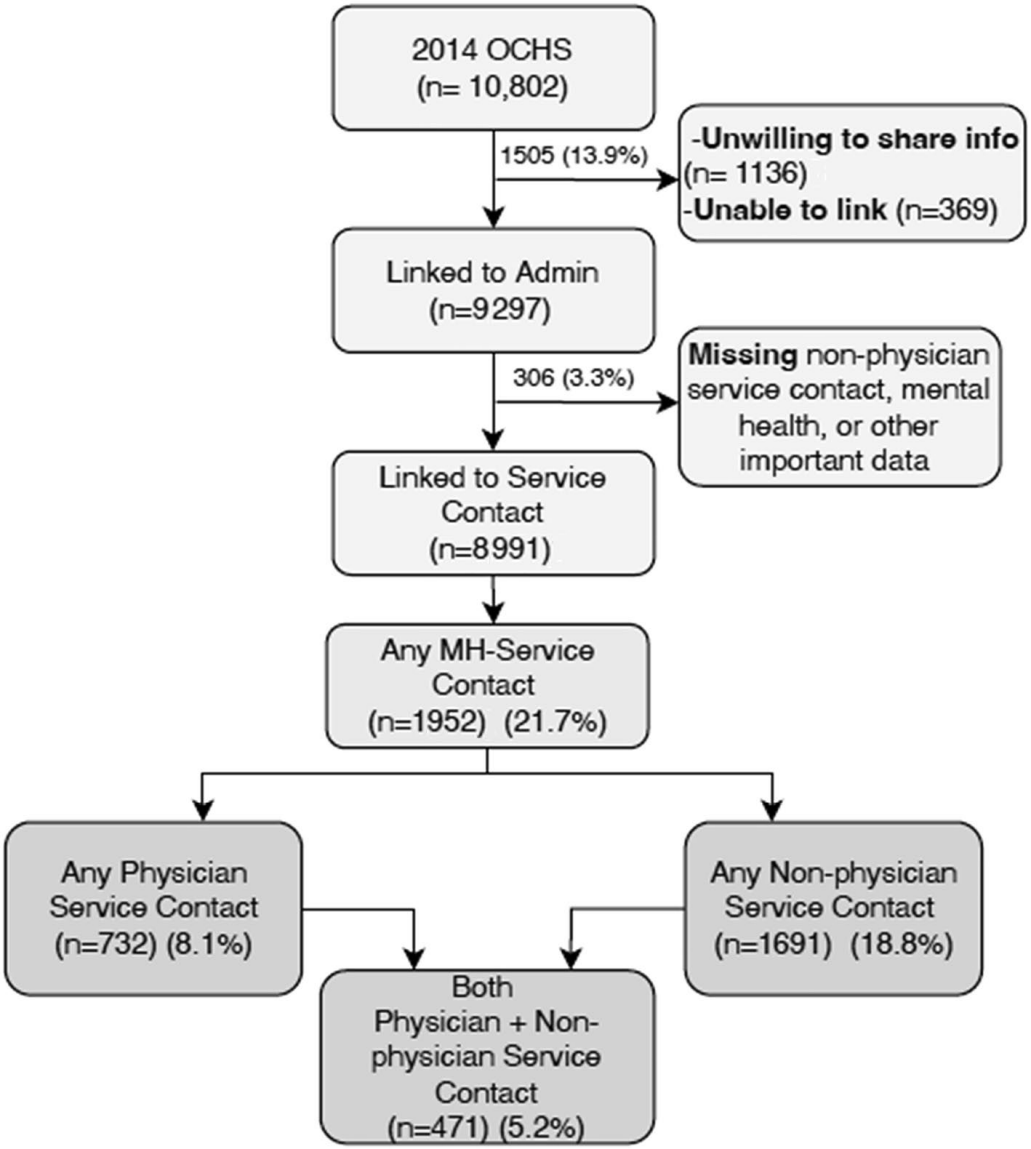
Flow chart of the data linkage and derivation of the study sample.

A total of 6537 households participated in the 2014 OCHS (50.8% response rate), including 10,802 children and youth aged 4 to 17 years. 89.5% of parents who completed the survey agreed to share their data for linkage (n = 9666) and 96.2% of those who agreed were successfully linked to administrative data (n = 9297). Three percent of the sample were missing service contact information, so a complete case analysis was conducted. This resulted in an analytic sample of 8991 (83.2% of full OCHS sample). Figure 1 provides a flow chart of the data linkage and derivation of the analytic sample. All analyses used sampling weights to generate estimates that are representative of the target population of children and youth in Ontario. Furthermore, to support the complex survey design, we applied mean bootstrap weights to produce accurate standard errors [11].

We estimated the prevalence of mental health related service contacts across the following types of professionals: (1) any physician services; (2) any non-physician services; (3) both physician and non-physician services; and (4) neither physician nor non-physician services overall and by socio-demographic groups. We present prevalence estimates and accompanying 95%CI for the socio-demographic characteristics and compared differences between groups using unadjusted Wald Chi-Squared tests. We estimated standardized mean dimensional measures of emotional, behavioural, and attention disorder symptoms and contacts with mental health related service groups. These analyses were stratified by age into children (age 4–11 years) and youth (age 12–17 years).

We assessed the socio-demographic and clinical correlates of mental health related service contact for physician and non-physician services. First, we compared children and youth contacting any mental health related service with those not contacting services. Second, among children and youth contacting services we compared characteristics of children and youth across the following groups (1) physician service vs non-physician service; (2) physician services vs both physician and non-physician services; (3) non-physician services vs both physician and non-physician services. We modeled these associations using standard and multinomial modified Poisson regression analyses to estimate prevalence ratios. For all analyses, alpha was set at 0.05. All analyses were conducted using STATA (version 14).

## Results

Our analytic sample includes 8991 children and youth, of which 4565 are male (50.8%) and 4426 are female (49.2%). The mean age of the sample was 10.6 (SD = 4.06) years. The 6-month prevalence of any mental health related service contact was 21.7% (n = 1952, 95% CI 19.6%, 23.4%), any physician service contact was 8.0% (n = 732, 95% CI 6.9%, 9.1%), any non-physician service contact was 18.8% (n = 1691, 95% CI 16.9%, 20.5%), and both physician and non-physician service contact was 5.2% (n = 471, 95% CI 4.3%, 6.1%) (see Fig. 1). Our findings indicate that most physician contacts were in outpatient settings (8% of the total sample) and there were few emergency department visits (0.3% of the total sample) and hospitalizations (0.1% of the total sample). The most frequent non-physician contacts were with teachers or other adults at school (13% of the total sample), followed by visits to CYMHS settings (7% of the total sample), visits to school guidance counsellors (6.2% of the total sample), visits to social workers (3.2% of the total sample), visits to psychologists (2.4% of the total sample), and visits to other type of counsellors (2.3% of the total sample) (see Additional file 4).

Among children ages 4–11 years, being male and living with one or no biological parents in the home was associated with significantly higher prevalence of physician, non-physician, and both service contacts. Low-income households had significantly more physician service contacts and contacts with both physician and non-physician services. Children with immigrant parent(s) had a significantly lower prevalence of non-physician service contact and of contacting both physician and non-physician services. In youth ages 12–17 years, living with one or no biological parents in the home was associated with significantly higher prevalence of physician, non-physician, and both service contacts. Youth with immigrant parent(s) had a lower prevalence of physician, non-physician, and both service contacts (see Table 1). We found the highest mean dimensional scores in children and youth receiving both physician and non-physician service contacts (see Table 1, Additional File 5).

**Table 1:** Prevalence of service contact % (95% CI) by varying socio-demographic and clinical characteristics of children and youth.

	Prevalence of service contact, % (95% CI)							
	Children (4–11 years) N = 5376				Children (12–17 years) N = 3615			
Variable	Physician (admin) (n = 410)	Non-physician (survey) (n = 1085)	Both (n = 272)	Neither (n = 4153)	Physician (admin) (n = 322)	Non-physician (survey) (n = 606)	Both (n = 199)	Neither (n = 2886)
Sex								
Male	9.24 (7.48, 11.01)	22.78 (19.90, 25.67)	6.54 (4.88, 8.21)	74.52 (71.62, 77.41)	9.98 (7.19, 12.76)	16.84 (13.72, 19.95)	5.26 (3.74, 6.78)	79.04 (75.36, 82.72)
Female	5.17 (3.69, 6.66)	17.88 (14.44, 21.33)	3.29 (2.06, 4.53)	80.24 (76.77, 83.71)	7.92 (6.23, 9.61)	16.29 (12.95, 19.62)	5.86 (3.82, 7.89)	81.06 (77.74, 84.36)
Chi Squared, P value	13.2*, p < 0.001	4.93*, p = 0.03	10.11*, p = 0.002	6.60*, p = 0.01	1.69, p = 0.19	0.05, p = 0.82	0.22, p = 0.64	0.67, p = 0.41
Number of biological parents in home								
Two	6.63 (5.18, 8.09)	18.82 (16.20, 21.44)	4.48 (3.20, 5.76)	79.02 (76.34, 81.69)	6.99 (5.46, 8.53)	13.58 (11.39, 15.76)	4.04 (2.97, 5.10)	83.47 (81.04, 85.89)
One or no bio-parents	10.33 (7.52, 13.14)	28.16 (23.59, 32.72)	7.34 (4.79, 9.88)	68.85 (64.28, 73.43)	15.7 (10.88, 20.44)	26.57 (21.08, 32.06)	10.69 (6.71, 14.67)	68.46 (62.00, 74.92)
Chi Squared, P value	5.54*, p = 0.019	14.07*, p < 0.001	4.14*, p = 0.042	15.73*, p < 0.001	17.88*, p < 0.001	24.12*, p < 0.001	19.02*, p < 0.001	25.19*, p < 0.001
Household poverty^a^								
Low-income	6.23 (4.78, 7.69)	20.06 (17.32, 22.81)	4.36 (3.08, 5.65)	78.06 (75.32, 80.79)	9.07 (7.17, 10.97)	16.69 (14.29, 19.09)	5.64 (4.13, 7.16)	79.87 (77.03, 82.72)
Not low-income	11.52 (8.73, 14.30)	21.79 (17.89, 25.67)	7.46 (5.46, 9.45)	74.15 (70.04, 78.26)	8.59 (5.93, 11.24)	15.98 (12.2, 19.80)	5.21 (3.07, 7.36)	80.64 (76.72, 84.57)
Chi Squared, P value	11.50*, p < 0.001	0.54, p = 0.46	6.79*, p = 0.009	2.64, p = 0.10	0.09, p = 0.76	0.10, p = 0.75	0.10, p = 0.76	0.11, p = 0.74
Immigrant parents								
Immigrant	6.28 (4.37, 8.19)	13.91 (11.32, 16.45)	3.04 (1.78, 4.29)	82.85 (80.03, 85.67)	5.22 (3.66, 6.77)	7.41 (5.46, 9.36)	2.79 (1.61, 3.98)	90.16 (88.08, 92.24)
Non-immigrant	7.98 (6.25, 9.71)	25.16 (22.01, 28.32)	6.38 (4.73, 8.02)	73.23 (70.12, 76.35)	11.82 (9.12, 14.52)	23.5 (20.30, 26.60)	7.65 (5.47, 9.84)	72.38 (68.69, 76.07)
Chi Squared, P value	1.48, p = 0.22	33.89*, p < 0.001	8.80*, p = 0.003	23.15*, p < 0.001	16.85*, p < 0.001	59.20*, p < 0.001	13.41*, p < 0.001	66.83*, p < 0.001
Urban–rural residency								
Large urban centre	7.17 (5.69, 8.66)	18.75 (16.1, 21.39)	4.96 (3.65, 6.27)	79.04 (76.33, 81.75)	8.52 (6.52, 10.53)	14.41 (12.02, 16.79)	4.66 (3.14, 6.17)	81.72 (78.72, 84.73)
Small-medium centre	7.19 (3.56, 10.82)	26.96 (20.59, 33.32)	5.35 (1.99, 8.71)	71.19 (64.84, 77.55)	8.51 (5.01, 12.02)	21.35 (14.69, 28.01)	4.66 (2.53, 6.79)	74.79 (68.19, 81.39)
Rural area	7.86 (3.26, 12.46)	20.08 (13.57, 26.59)	4.41 (2.02, 6.79)	76.46 (69.34, 83.58)	11.73 (6.95, 16.49)	21.18 (16.13, 26.23)	10.92 (6.11, 15.73)	78.01 (72.87, 83.16)
Chi Squared, P value	L-S 0.00, p = 0.99 L-R 0.08, p=0.77 S-R 0.04, p=0.83	L-S 6.30*, p = 0.012 L-R 0.14, p=0.71 S-R 2.20, p=0.14	L-S 0.04, p = 0.83 L-R 0.15, p=0.69 S-R 0.19, p=0.66	L-S 5.47*, p = 0.019 L-R 0.46, p=0.49 S-R 1.12, p=0.29	L-S 0.00, p = 0.99 L-R 1.58, p=0.21 S-R 1.20, p=0.27	L-S 4.62*, p = 0.011 **L-R 6.22, p=0.013** S-R 0.00, p=0.97	L-S 0.00, p = 0.99 **L-R 8.43, p=0.004** **S-R 6.61, p=0.010**	L-S 4.32*, p = 0.038 L-R 1.46, p=0.23 S-R 0.56, p=0.46
Disorder (parent report)^b^	Mean Dimensional Scores (95%CI)							
Internalizing	0.70 (0.41, 0.99)	0.71 (0.55, 0.86)	1.09 (0.71, 1.48)	− 0.19 (− 0.24, − 0.15)	1.31 (1.00, 1.61)	1.17 (0.94, 1.39)	1.79 (1.42, 2.17)	− 0.18 (− 0.23, − 0.12)
Externalizing	0.83 (0.61, 1.05)	0.68 (0.56, 0.81)	1.27 (1.00, 1.53)	− 0.19 (− 0.22, − 0.15)	0.76 (0.51, 1.04)	0.93 (0.70, 1.16)	1.09 (0.71, 1.48)	− 0.18 (− 0.22, − 0.13)
ADHD	1.20 (0.96,1.44)	0.91 (0.78, 1.04)	1.69 (1.40, 1.99)	− 0.12 (− 0.16, − 0.08)	0.77 (0.52, 1.02)	0.62 (0.46, 0.78)	0.97 (0.66, 1.28)	− 0.32 (− 0.36, − 0.27)

^a^Household poverty determined using the low-income measure, which is based on the 2013 before tax-cut-offs in Canada [21]

*p<0.05

^b^Clinical characteristics are represented using standardized mean dimensional measures of mental health where the average in the population is equal to 0 and 1 is indicative of one standard deviation increase in symptoms of that disorder category

After adjusting for total mental health symptom ratings and socio-demographic characteristics, older youth aged 12–17 years (PR: 0.84, 95% CI 0.73, 0.97) and children and youth with immigrant parent(s) (PR: 0.65, 95% CI 0.55, 0.75) were less likely to have mental health related service contacts. Children and youth with one or no biological parents in the home (PR: 1.31, 95% CI 1.10, 1.55), compared to both biological parents in the home, were more likely to have any mental health related service contacts. Positive associations between internalizing (PR: 1.31, 95% CI 1.24, 1.38) and ADHD (inattentive and hyperactivity) symptoms (PR: 1.28, 95% CI 1.21, 1.35) and any mental health related service contacts were also found. Our findings also indicate that among those with service contacts, older youth aged 12–17 years (PR: 3.02, 95% CI 1.79, 5.07) and children and youth with immigrant parent(s) (PR: 2.23, 95% CI 1.16, 4.28) were more likely to have physician service contacts alone, compared to non-physician services alone. Children and youth with increased symptoms of internalizing (PR: 0.75, 95% CI 0.59, 0.94) and externalizing disorders (PR: 0.68, 95% CI 0.53, 0.88), were less likely to have physician service contacts alone, compared to non-physician services alone. Similar trends were observed when comparing children and youth physician and non-physician service contacts alone, compared to both physician and non-physician services (see Table 2).

**Table 2:** Modified Poisson regression exploring the socio-demographical and clinical correlates of mental health related service contacts across provider types.

Sample/model	Total sample/non-multinomial	Among children and youth with service contacts/multinomial		
Outcomes	Any MH service contact Physician or non-physician (n = 1952)	Physician Only (n = 261) vs Non-physician only (n= 1220)	Physician only (n = 261) **vs** both physician + non-physician (n = 471)	Non-physician only (n = 1220) **vs** both physician + non-physician (n = 471)
Variable	Adjusted for All Factors PR^a^ (95% CI)	Adjusted for All Factors PR^a^ (95% CI)	Adjusted for All Factors PR^a^ (95% CI)	Adjusted for All Factors PR^a^ (95% CI)
Age				
4–11 years	Ref.	Ref.	Ref.	Ref.
12–17 years	0.84* (0.73, 0.97)	3.02* (1.79, 5.07)	1.94* (1.05, 3.59)	0.64* (0.44, 0.93)
Sex				
Female	Ref.	Ref.	Ref.	Ref.
Male	1.08 (0.93, 1.25)	1.38 (0.81, 2.36)	1.10 (0.59, 2.05)	0.79 (0.53, 1.19)
Number of biological parents in home				
Two	Ref.	Ref.	Ref.	Ref.
One or no biological parents	1.31* (1.10, 1.55)	0.85 (0.41, 1.77)	0.69 (0.30, 1.61)	0.82 (0.53, 1.27)
Household poverty				
Not low-income	Ref.	Ref.	Ref.	Ref.
Low-income	0.95 (0.82, 1.09)	1.82 (0.91, 3.63)	1.46 (0.71, 3.02)	0.80 (0.52, 1.24)
Immigrant Backgr.				
Non- Immigrant	Ref.	Ref.	Ref.	Ref.
Immigrant	0.65* (0.55, 0.75)	2.23* (1.16, 4.28)	2.17* (1.06, 4.43)	0.97 (0.60, 1.57)
Urban–rural residency				
Large urban centre	Ref.	Ref.	Ref.	Ref.
Small-medium centr	1.09 (0.92, 1.29)	0.83 (0.38, 1.83)	1.44 (0.53, 3.91)	1.73 (0.91, 3.31)
Rural area	1.05 (0.85, 1.29)	0.79 (0.26, 2.42)	0.47 (0.15, 1.49)	0.59 (0.34, 1.05)
Internalizing^b^	1.31* (1.24, 1.38)	0.75* (0.59, 0.94)	0.59* (0.45, 0.78)	0.79* (0.68, 0.93)
Externalizing^b^	1.06 (0.99, 1.13)	0.68* (0.53, 0.88)	0.71* (0.53, 0.94)	1.04 (0.91, 1.19)
ADHD^a^	1.28* (1.21, 1.35)	1.15 (0.95, 1.39)	0.79 (0.62, 1.01)	0.69* (0.57, 0.84)

^a^PR prevalence ratio

*p < 0.05

^b^Parent report

## Discussion

This work represents one of the first studies in North America to use population level survey data linked with health administrative data to estimate the correlates of mental health related service contacts among children and youth across sectors, adjusting for factors relating to mental health need. As such, our findings provide unique insight into disparities in mental health related services across sectors among children and youth, in which adjustment of factors related to mental health need is essential.

Our findings suggest that 1 in 5 children and youth had a mental health related service contact in the six-month period prior to completing the OCHS survey. Our estimate is comparable to prior work from the US, which estimated that the prevalence of any mental health related service contact was 16% [21]. We found that mental health related service contacts vary by (i) provider, (ii) by clinical factors, including symptom ratings, disorder class, and (iii) by socio-demographic characteristics including age, sex, family structure, and immigrant parent(s). Unsurprisingly, our results suggest that higher symptom ratings were associated with a higher likelihood of contacting help across providers. In particular, children and youth contacting both physician and non-physician services had higher mean symptom ratings of mental disorders across all disorder classes. Furthermore, increasing symptoms of internalizing and externalizing disorders were both negatively associated with contacting physician services alone, compared to both physician and non-physician services. While consistent with prior work [21], which suggests youth with higher symptom ratings are more likely to contact multiple sectors, this important finding highlights the increased complexity in patterns of service contacts among children and youth with the highest symptom ratings, who may have the greatest mental health service needs. As such, the coordination of care across and within sectors are critical components of mental health services for children and youth [21]. Further research is needed to better understand coordination of care across sectors and models of stepped care, where children and youth with the greatest needs receive more specialized care [31].

Our results indicate that the greatest disparities in mental health contacts may exist for children and youth with immigrant parent(s). After adjusting for total mental health symptom ratings and other socio-demographic characteristics, children and youth with immigrant parent(s) had lower mental health related service contacts, which may reflect disparities in service delivery, rather than variation in the clinical profile of various groups [1, 32, 33]. Furthermore, we found children and youth with immigrant parent(s) were more likely to have contacted physician services alone, compared to non-physician services alone or both physician and non-physician services. These findings build on prior evidence suggesting immigrant youth are more likely to have a first point of mental health contact be an emergency department [34]. As we were able to include non-physician contacts in our analysis, our findings highlight the need to not only reduce barriers to care in outpatient physician settings, but also in non-physician settings, which may help contribute to identifying and treating mental health problems earlier, prior to crisis [34]. Our findings also suggest there is similarly a need to reduce barriers to care for older youth ages 12 to 17 years, who experience lower mental health related service contacts, compared to children ages 4 to 11 years. This finding aligns with prior work describing an increase in barriers to mental health related services for aging adolescents and transitional aged youth [35]. Future research is needed to better describe the types of barriers to care older adolescents are experiencing in Canada.

Our findings suggest an important correlate of mental health related service contact is living with one or no biological parents. This finding reflects the importance of family context in understanding mental health related service contact, and is consistent with prior survey research, however, is missing from administrative data sources, as this information is not regularly captured [1, 36–38]. Children and youth living with one or no biological parents may be more likely connected with the foster care system and other social services, which may facilitate mental health related service contacts. Further research is needed to better understand where and in which settings lone parent families are accessing mental health related services.

Our findings indicate comprehensive characterization of mental health related service contacts among children and youth relies on the measurement of both physician and non-physician services. Our prevalence estimates of service contacts across sectors/professionals demonstrates that non-physician service contacts are much more common. As such, our findings highlight the limitations of relying on the use of health administrative data in isolation, which is currently limited to physician contacts. The use of data linkage has increased both the breadth of mental health related service contacts and the depth in which children and youth can be characterized. As such, our findings highlight the strength of using data linkage to study the correlates of child and youth mental health related service contact across providers, compared to relying on individual data sources on their own [8]. Going forward, we believe there is a need to better understand the types of treatments children and youth are receiving from various service providers to further characterize children and youth receiving mental health related services.

## Limitations

Our results may not be generalizable outside of Ontario due to the provincial variation in mental health service provisions and approach to diagnostic coding [40]. Another limitation is that administrative data is a product of physician billing codes. As such, thresholds for diagnosis will vary between physicians and settings [41]. Our findings may be subject to information biases, including recall bias, telescoping bias, and potential differences among participants with, versus without, complete data. Such biases may influence the observed associations [13–15, 42]. It is important to note that while our non-participation bias and an unwillingness to share linked data may have impacted our findings. Specifically, families of children with more severe symptoms of mental disorders may be less likely to participate in a survey and children and youth, which may not be addressed by survey weighting conducted by Statistics Canada. Furthermore, the sampling frame did not include Indigenous children living on reserve, those who are homeless, and those in the child welfare or youth justice systems, who may be at greater risk for mental health related concerns. As such, our findings may not be generalizable to children and youth with the most severe mental disorders [42]. Our approach to classifying mental health related service contacts is broad and inclusive and should not be equated with receipt of mental health treatment. Our classification reflects the data source and methodology from which they are derived and our sample distributions, which have limited our ability to differentiate between various service settings including differences between outpatient and acute care (emergency department, hospital) settings. As such, to better contextualize mental health related services that children and youth are receiving, we believe going forward, there is a need to better understand the types of treatments children and youth are receiving from various service providers. It is important to note that while we did not identify significant variation in mental health related services by rurality, evidence suggests there exists regional variation in service delivery in Ontario [10]. Furthermore, there are a number of area-level indicators we were unable to adjust for, which may have led to residual confounding; including area-level deprivation and indicators of the availability of healthcare and school related mental health services.

## Conclusion

Our findings indicate important variation in mental health related service contacts by provider, clinical factors including symptom ratings and disorder class, and socio-demographic characteristics including age, immigrant background, and family structure. Furthermore, the coordination of care across and within sectors are critical components of mental health services for children and youth. This work suggests the greatest disparities in mental health related services may exist for children and youth with immigrant parent(s). Targeted outreach efforts to reduce barriers to care are required. This study highlights the strengths of using data linkage to study the correlates of child and youth mental health related service contacts across providers and sectors. Investment should be made to expand available population-level data linkages for children and youth, as these linkages offer strong platforms for improving our understanding of mental health service delivery.

## Supplementary Information

**Additional file 1: **Included databases.

**Additional file 2: **RECORD guidelines.

**Additional file 3: **Coding for mental healthrelated service contacts.

**Additional file 4: **Mental health related service contact estimates for children and youth.

**Additional file 5: **Standardized mean dimensional measures of mental health of children and youth with varying service contacts. Average in the population is equal to 0 and 1 is indicative of one standard deviation increase in symptoms of that disorder category.Standardized mean dimensional measures of mental health of children and youth with varying service contacts. Average in the population is equal to 0 and 1 is indicative of one standard deviation increase in symptoms of that disorder category.

## Data Availability

Data access to the 2014 Ontario Child Health Study is available through Statistics Canada Research Data Centres. Data access to the linked dataset will not be shared due to data sharing agreements with Statistics Canada and the Ministry of Health in Ontario.

## Abbreviations

OCHS: Ontario Child Health Study
OCHS-EBS: Ontario Child Health Study Emotional Behavioural Scales
MOHLTC: Ministry of Health and Long-Term Care
OHIP: Ontario Health Insurance Program
RECORD: Reporting of Studies Conducted Using Routinely Collected Data
DSM-5: Diagnostic and Statistical Manual of Mental Disorders
MDD: Major Depressive Disorders
GAD: Generalized Anxiety Disorder
SP: Social Anxiety Disorder/Social Phobia
SAD: Separation Anxiety
ODD: Oppositional-Defiant Disorder
CD: Conduct Disorder
ADHD: Attention-Deficit Hyperactivity Disorder
MINI-KID: Mini International Neuropsychiatric Interview for Children and Adolescents
PMK: Persons Most Knowledgeable
CIHR: Canadian Institutes of Health Research
