# Supplementary material for: Characterizing mental health related service contacts in children and youth: a linkage study of health survey and administrative data
Source: Child Adolesc Psychiatry Ment Health. 2022 Jun 21;16:48. doi: 10.1186/s13034-022-00483-w (PMC9215063; doi:10.1186/s13034-022-00483-w)
Supplement: Supplementary file 1 — Additional file 1: Included databases. [file 13034_2022_483_MOESM1_ESM.docx]

**S1:** Included Databases.

| Database | Description |
| --- | --- |
| Claims History Database (CHD) | Physician outpatient visits including visits to family physicians, pediatricians, and psychiatrists. |
| Discharge Abstract Database (DAD) | Inpatient hospitalizations.^(34)^ |
| National Ambulatory Care Reporting System (NACRS) | Visits to the emergency department.^(35)^ |
| Registered Persons Database (RPDB) | Contains information on people registered under OHIP. Used to determine OHIP eligibility. |
| Client Agency Program Enrolment Database (CAPE) | Contains information on people registered with a primary care organization. Used to determine OHIP eligibility. |
