# Supplementary material for: Characterizing mental health related service contacts in children and youth: a linkage study of health survey and administrative data
Source: Child Adolesc Psychiatry Ment Health. 2022 Jun 21;16:48. doi: 10.1186/s13034-022-00483-w (PMC9215063; doi:10.1186/s13034-022-00483-w)
Supplement: Supplementary file 3 — Additional file 3: Coding for mental healthrelated service contacts. [file 13034_2022_483_MOESM3_ESM.docx]

**S3:** Coding for Mental Health Related Service Contacts.

| **Physician Service Contacts Derived from Administrative Data.** | | |
| --- | --- | --- |
| *Disorders included in mental health related-service contacts variable* | *NACRS & DADS (ICD10)* | *OHIP (ICD9)* |
|  | **F10-19, F55** Substance-related disorders  **F20 (excluding F20.4), F22-25, F28, F29, F53.1** Schizophrenia  **F21, F60-62, F68, F69, F80-F84, F88-92, F94, F95, F98** Neurodevelopmental and personality disorders  **F30-F39**: Mood disorders  **F40-48**: Anxiety disorders  **F50.0-50.3, F50.8, F50.9** Eating disorders  **F90** Hyperkinetic disorders  **F92** Mixed disorders of conduct and emotions  **F93** Emotional disorders with onset specific to childhood  **F98.8** Other specified behavioural and emotional disorders with onset usually occurring in childhood and adolescence  **F98.9** Unspecified behavioural and emotional disorders with onset usually occurring in childhood and adolescence  **F99** Mental disorder, not otherwise specified | **291, 292, 299** Alcohol, drug or other psychoses  **295-298** Psychotic disorders  **300** Neurotic disorders (includes reactive depression)  **301, 302, 306, 309** Non-psychotic disorders  **303, 304** Substance use disorders  **307** Habit spasms, tics, anorexia nervosa, sleep disorders  **311** Depressive disorder, not elsewhere classified (Include: depressive disorder NOS, depressive state NOS, depression NOS)  **313** Disturbance of emotions specific to childhood and adolescence  **314** Hyperkinetic syndrome of childhood  **315** Developmental delay  **316** Psychic factors associated with diseases classified elsewhere  **897-902, 904-906, 909** Social problems |
| **Non-physician Service Contacts Derived from 2014 OCHS** | | |
| *Contact Points for Community Service Providers* | *Provider/Service* | *Guiding Question from the OCHS* |
|  | CYMHS Agencies* | MCYS MH agency visit for MH concerns (code yes if any response option is present) |
|  | Psychologists | Seen a care provider about MH concerns-Psychologist |
|  | Social Worker | Seen a care provider about MH concerns-Social worker |
|  | Some Other Type of Counsellor | Seen a care provider about MH concerns-Other type of counsellor |
|  | School Guidance Counsellor | Seen a care provider about MH concerns-School guidance counsellor |
|  | Teacher or other adult at School | Other help-teacher or other adult at school |
|  | School individual or group counselling | School individual or group counselling |
| * While CYMHS agencies provide a combination of physician and non-physician-based services, we’ve only including agencies that are not funded from the Ministry of Health and Long Term Care (MOHLTC) in attempts to isolate self-reported contacts with non-physician based services. | | |
