# Supplementary material for: Characterizing mental health related service contacts in children and youth: a linkage study of health survey and administrative data
Source: Child Adolesc Psychiatry Ment Health. 2022 Jun 21;16:48. doi: 10.1186/s13034-022-00483-w (PMC9215063; doi:10.1186/s13034-022-00483-w)
Supplement: Supplementary file 4 — Additional file 4: Mental health related service contact estimates for children and youth. [file 13034_2022_483_MOESM4_ESM.docx]

**S4:** Mental health related service contact estimates for children and youth.

| Variable | Provider | Prevalence of Mental Health Related Contact % (95%CI) |
| --- | --- | --- |
| Physician Service (Admin) | Hospital | 0.1 (0.03,0.2) |
|  | Emergency Physician | 0.3 (0.2,0.4) |
|  | OHIP (Pediatrician, Psychiatrist, Family doctor and other Physician Specialist) | 8.0 (6.9,9.1) |
| Non-Physician Service (Survey) | CYMHS agency contact | 7.0 (5.8,8.1) |
|  | Psychologists | 2.4 (1.8,3.0) |
|  | Social workers | 3.2 (2.5,4.0) |
|  | Other type of counsellor | 2.3 (1.7,3.0) |
|  | School guidance counsellor | 6.2 (5.1,7.4) |
|  | Teacher or other adult at school | 13.0 (11.4,14.7) |
