# Supplementary material for: Characterizing mental health related service contacts in children and youth: a linkage study of health survey and administrative data
Source: Child Adolesc Psychiatry Ment Health. 2022 Jun 21;16:48. doi: 10.1186/s13034-022-00483-w (PMC9215063; doi:10.1186/s13034-022-00483-w)
Supplement: Supplementary file 5 — Additional file 5: Standardized mean dimensional measures of mental health of children and youth with varying service contacts. Average in the population is equal to 0 and 1 is indicative of one standard deviation increase in symptoms of that disorder category.Standardized mean dimensional measures of mental health of children and youth with varying service contacts. Average in the population is equal to 0 and 1 is indicative of one standard deviation increase in symptoms of that disorder category. [file 13034_2022_483_MOESM5_ESM.docx]

**S5:** Standardized mean dimensional measures of mental health of children and youth with varying service contacts. Average in the population is equal to 0 and 1 is indicative of one standard deviation increase in symptoms of that disorder category.

| Mean Dimensional Scores (95%CI) | | | | |
| --- | --- | --- | --- | --- |
|  | Children (12-17 years) Youth Report (N=3458) | | | |
| Disorder | Physician (Admin) (n=300) | Non-Physician (Survey) (n=567) | Both  (n=184) | Neither  (n=2775) |
| Internalizing | 0.82  (0.55, 1.08) | 0.60  (0.41, 0.79) | 0.98  (0.62, 1.34) | -0.13  (-0.18, -0.07) |
| Externalizing | 0.71  (0.45, 0.96) | 0.45  (0.29, 0.61) | 0.81  (0.43, 1.19) | -0.13  (-0.19, -0.07) |
| ADHD | 0.77  (0.53, 1.01) | 0.47  (0.32, 0.62) | 0.86  (0.57, 1.16) | -0.13  (-0.19, -0.05) |
